# Genetic, evolutionary and plant breeding insights from the domestication of maize

**DOI:** 10.7554/eLife.05861

**Published:** 2015-03-25

**Authors:** Sarah Hake, Jeffrey Ross-Ibarra

**Affiliations:** Plant Gene Expression Center, US Department of Agriculture-Agriculture Research Service, Albany, United States and Department of Plant and Microbial Biology, University of California, Berkeley, Berkeley, United States; Department of Plant Sciences, Center for Population Biology and Genome Center, University of California, Davis, Davis, United States

**Keywords:** the natural history of model organisms, maize, teosinte, domestication, maize

## Abstract

The natural history of maize began nine thousand years ago when Mexican farmers started to collect the seeds of the wild grass, teosinte. Invaluable as a food source, maize permeated Mexican culture and religion. Its domestication eventually led to its adoption as a model organism, aided in large part by its large chromosomes, ease of pollination and growing agricultural importance. Genome comparisons between varieties of maize, teosinte and other grasses are beginning to identify the genes responsible for the domestication of modern maize and are also providing ideas for the breeding of more hardy varieties.

**DOI:** http://dx.doi.org/10.7554/eLife.05861.001

## Introduction

**Figure 1.**
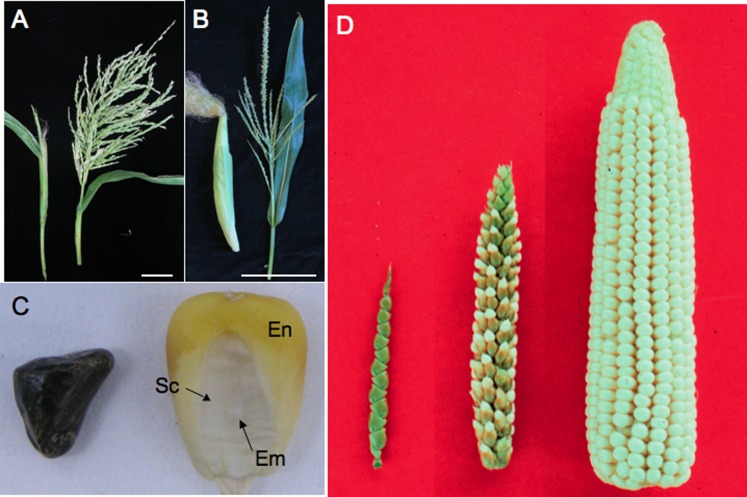
Teosinte compared to maize. (**A**) A teosinte female inflorescence (left), which arises as a secondary branch from tillers, and tassel (right). (**B**) An ear (left) and tassel (right) of maize. Size bar in **A** and **B** is 10 cm. (**C**) Teosinte kernel (left) and maize kernel (right). The teosinte kernel is hidden by hardened glumes (see Glossary). The maize kernel is exposed and reveals the endosperm (En) and embryo (Em). The embryo is surrounded by the scutellum (Sc), the nutritive tissue of the cotyledon. (**D**) A comparison of teosinte on the left, maize on the right and the F1 of maize and teosinte in the middle. Image credits: (**D**) John Doebley, Department of Genetics, University of Wisconsin–Madison; all other images, Sarah Hake. **DOI:** http://dx.doi.org/10.7554/eLife.05861.002

As one of the world's most important crops, maize (corn) needs little introduction. What is less well appreciated is the story of its remarkable transformation (Figure 1). Genetic data point to the tropical Balsas river valley in Mexico as the site where maize (*Zea mays* ssp. *mays*) was domesticated from teosinte (*Zea mays* ssp. *parviglumis*) (Matsuoka et al., 2002; van Heerwaarden et al., 2011). Archeological data support this location and also suggest that squash may have been domesticated at the same time (Piperno et al., 2009). Some think maize was first collected for the fermentable sugars in its stalk (Iltis, 2000; Smalley and Blake, 2003), but more likely it was for the storable starch in its seed.

The word teosinte is derived from ‘teocintli’—‘teotl’ meaning sacred and ‘cintli’ meaning dried ear of corn—from the indigenous Nahuatl language. We use the word teosinte to refer to all the wild species of *Zea* that are native to Mexico and Central America. Teosinte sows its seeds widely. In addition to dispersing pollen in the wind, the kernels fall from the plant and, if eaten, are carried to other locations in fecal matter, thanks to the indigestible fruitcase (see Box 1 for a glossary of specialist terms used in this article). The domestication of maize kept the wind-born pollen of teosinte, but changed other traits, improving its utility for human consumption (Doebley, 2004). The teosinte fruitcase, full of silica and lignin, became softer (Figure 1C), allowing humans to grind its kernels for food. The branch holding the kernels (cob) grew in girth, increasing the kernel row number from 2 to 20, or more (Figure 1D). Kernels no longer self-dispersed but were held tight on the cob, requiring the intervention of humans to sow seeds. Finally, the long branches shortened, but kept the leaves along the branch. These ‘husk leaves’ keep birds, insects and other pests from eating the kernels.

10.7554/eLife.05861.003

Box 1. **Glossary**

**Axil:** Where a leaf joins the stem. Buds form in the axil.

**C4 photosynthesis:** A more efficient form of photosynthesis in which CO_2_ is fixed into a four-carbon sugar.

**Fruitcase:** Maternal tissue that surrounds the maize or teosinte kernels.

**Gametophyte:** The multicellular haploid structure from which plant gametes derive.

**Glume:** The leaf that subtends the flower, it is usually sterile (no buds in its axil).

**Inbreeding depression:** Reduced biological fitness caused by inbreeding.

**Landraces:** Corn varieties that are maintained by open pollination and not by controlled crosses.

**Pistil:** Female part of a flower.

**Polar nuclei:** The two nuclei of the large central cell of the female gametophyte; they fuse with a nucleus of one sperm cell to generate the triploid endosperm.

**Predictive breeding:** Breeding strategies that utilize statistical models to predict phenotype from genotype information. They potentially save considerable time and money by reducing the number of generations of field testing needed.

**Quantitative trait:** Traits that are polygenic, also called complex traits.

**Quantitative trait locus:** A genomic region statistically associated with phenotypic differences of a quantitative trait.

**Recombinant inbred population:** A population of homozygous individuals obtained by the repeated self crossing of an F2 hybrid, consisting of ∼50% of each parental genome in different combinations. These lines are often used for mapping complex traits.

**Scutellum:** Part of the cotyledon that absorbs nutrients from the endosperm for the embryo.

**Shoot apical meristem:** A group of totipotent stem cells that produce leaves and branches.

**Transposable elements:** Genetic elements that can move from one chromosomal location to another.

**Vegetative cell:** The male gametophyte undergoes two divisions that produce two sperm cells and the vegetative cell. The sperm cells are contained within the vegetative cell.

**DOI:** http://dx.doi.org/10.7554/eLife.05861.003

**Figure 2.**
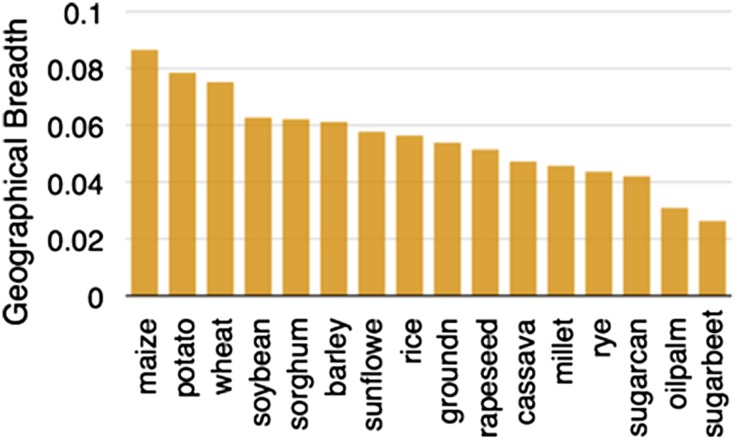
Geographic breadth of the world's 16 staple crops, expressed in percent of 5′ grid cells in which each crop is cultivated. Data are from (Monfreda et al., 2008), downloaded from earthstat.org. **DOI:** http://dx.doi.org/10.7554/eLife.05861.004

From the Balsas region of Mexico, maize spread north and south (Matsuoka et al., 2002), adapting to very different environmental conditions. In a testament to its adaptability, maize is now found growing across a wider area than any other major crop (Figure 2). Cobs of corn found in archeological sites suggest that major morphological changes had occurred by 6500 years ago (Piperno and Flannery, 2001). Pollen flow between domesticated maize and other teosinte taxa contributed to some of its diversification (van Heerwaarden et al., 2011), and local adaptation and trade continue to shape maize genetic diversity (Mercer et al., 2008; Ruiz Corral et al., 2008). Maize varieties in Mexico can often be distinguished by kernel or cob traits (Vielle-Calzada and Padilla, 2009) but genetic diversity within these open-pollinated populations remains high (Pressoir and Berthaud, 2004).

Darwin was the first to recognize inbreeding depression (see Glossary) in maize, a critical component of maize breeding. He noted a reduced size in self-pollinated corn and the improved size and vigor, or heterosis, of cross-pollinated corn (Darwin, 1876). His observations were extended by George Harrison Shull in 1908, who made inbreds by self-pollinating maize plants for multiple generations before crossing them together to make hybrids (Shull, 1908). Today, of course, hybrid maize is the predominant form grown in industrial agriculture (Duvick, 2001), with traditional open-pollinated landraces (see Glossary) primarily limited to small scale or subsistence farming.

## The lifecycle of maize

**Figure 3.**
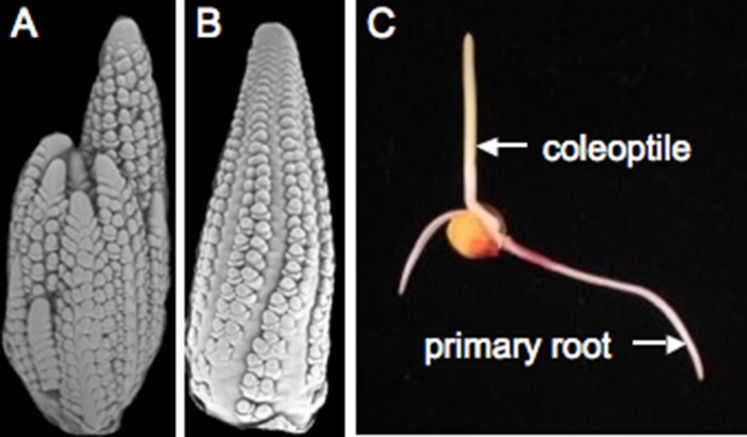
Maize reproduction. (**A**) Scanning electron microscopy (SEM) image of a tassel primordium. (**B**) SEM of an ear primordium. (**C**) A germinated maize seedling. Image credits: Sarah Hake. **DOI:** http://dx.doi.org/10.7554/eLife.05861.005

Teosinte is sensitive to day length and only makes flowers when days are short (Emerson, 1924). An important step in breeding maize for higher latitudes involved overriding this environmental control, creating maize plants that flower independently of day length. The maize shoot apical meristem (see Glossary) initiates a genetically determined number of leaves before it transitions to become the male reproductive structure, known as the tassel. Buds are found in the axils (see Glossary) of most leaves—the lower buds become tillers, which are long branches, resembling the main stem. The buds above the tillers will develop into the ears, which hold the kernels. During early development, the ear and tassel primordia appear similar to each other (Figure 3A,B). Sex determination genes initiate a program that blocks development of female reproductive parts in the male flowers of the tassel and male reproductive parts in the female flowers of the ear (Cheng et al., 1983). Interestingly, the tassel of a tiller is often partially feminized. While teosinte plants have multiple tillers, ensuring a supply of pollen over many days, modern maize often has a single tassel and lacks tillers entirely. Maize hybrids are created by planting two distinct inbreds in alternate rows and detasseling one inbred, ensuring that the ears on detasseled plants are cross-pollinated and thus hybrid.

As with other flowering plants, the male sperm is carried in the pollen. Following meiosis, the microspore undergoes two mitotic divisions, generating two sperm cells contained within a vegetative cell (see Glossary). The vegetative nucleus does the work of bringing the sperm cells to the female egg. The pollen germinates on silks, the extended pistils (see Glossary) of the female flower, and grows a tube inside the silk. Once the pollen tube reaches the plant's ovule, one sperm will fertilize the haploid egg cell, creating the diploid embryo, and the other sperm will fertilize the central cell of the female gametophyte, which has two polar nuclei (see Glossary). The resulting triploid cell develops into the endosperm, a sacrificial tissue that feeds the growing embryo.

Once the kernel, containing the double fertilization event, matures, it can be planted or stored for years in a dry state. The root emerges first, growing towards gravity. The shoot grows upward, supported by the nutrients of the endosperm (Figure 3C). Maize is a monocotyledonous plant, meaning it produces one embryonic leaf (as compared to another model plant *Arabidopsis thaliana*, which is a dicotyledonous plant). The cotyledon equivalent for maize is the coleoptile, which is connected to the nutritive scutellum (see Glossary). Inside the tubular coleoptile are sequentially younger leaves that arise from a shoot apical meristem.

## Strengths as a model organism

Geneticists began collecting, describing and distributing maize mutants in the early 1900's (see references in Emerson et al., 1935). They took advantage of a number of features that make maize a valuable resource for genetic research (Box 2). Dozens of genes affect the texture of the endosperm and the color of its outermost layer, the aleurone, providing easily visible and eye-catching information about the genotype of the next generation. The large, distinctive chromosomes of maize further paved the way for a number of major discoveries, including linking genetic recombination to physical exchange of chromosomal segments (Creighton and McClintock, 1931). The copious amounts of pollen maize plants produce can be easily gathered and mutagenized with chemicals to create new mutant plants, allowing researchers to easily screen for even lethal mutations (Neuffer, 1993; Candela and Hake, 2008). Transposable elements (see Glossary), first described by Barbara McClintock (McClintock, 1950), continue to be an important tool for cloning genes based on mutant phenotype, and their use has provided novel insights into epigenetics (Lisch, 2012), such as the relationship between transposon activity and DNA methylation (Chandler and Walbot, 1986).

10.7554/eLife.05861.006

Box 2. **The strengths of maize as a model organism.**

Many features make maize a valuable model for genetic research (Nannes and Dawe, 2015).

- Separate male and female flowers that allow easily controlled crosses.
- Hundreds of large seeds held in rows, making it easy to see rare events in kernel color or composition and to deduce segregation ratios.
- The availability of developmental series of meristems in the ear and tassel.
- The abnormal B chromosomes that undergo non-disjunction at meiosis; they are highly reduced but still have functional centromeres. B-A chromosome translocations facilitate studies of gene dosage (Birchler and Hart, 1987) and provide insights into mini-chromosomes (Birchler, 2014).
- The ease of creating controlled crosses, making maize the premier system for studying the genetic basis of heterosis (hybrid vigor); heterosis is key to its success as an agricultural crop.
- The availability of color genes to study paramutation (Hollick, 2012). Maize is an important organism for studying paramutation, which occurs when one allele heritably affects the gene regulation of another allele in *trans*; it also occurs in *Drosophila* and mouse.
- The existence of multiple populations of induced mutations for genetic analysis.
- The duplication and subsequent fractionation of the maize genome, along with its recent divergence from sorghum, provide ample material for studies of genome evolution.
- Years of breeding trials and experiments provide many examples of experimental evolution, which can now be analyzed using genomic tools.

**DOI:** http://dx.doi.org/10.7554/eLife.05861.006

## The position of maize in the grass family

Maize and teosinte are members of the grass family Poaceae, which includes more than 10,000 species (Kellogg, 1998), including the major cereals, such as wheat and rice. The subfamily that includes Zea is Panicoideae (3236 species), which also includes C4 energy crops (see Glossary) such as switchgrass, sorghum, miscanthus and the recently sequenced model, Setaria (Grass Phylogeny Working Group II, 2012). Wheat, barley, and the model Brachypodium are in the Pooideae subfamily (3850 species), while rice, a well-studied model, as well as an important crop, is in the Ehrartoideae (111 species) (Grass Phylogeny Working Group II, 2012). The latter two subfamilies contain only C3 grasses.

Despite the difference of more than 60 million years of evolution, studies of grass genomes have revealed that significant portions of their chromosomes share the same gene arrangement (Moore et al., 1995). This discovery provides an opportunity to isolate genes based on common positions of shared markers. With the ever-increasing number of sequenced grass genomes, such as rice, sorghum, Brachypodium, Setaria and maize (https://genomevolution.org/wiki/index.php/Sequenced_plant_genomes#Grasses), researchers can now take advantage of comparative genomics to identify orthologous genes and regulatory elements that are likely to be important for biological processes (Lin et al., 2012).

## Identifying domestication genes

George Beadle was the first to cross maize with teosinte and examine the F2 progeny (Beadle 1972). Based on the frequency of maize- or teosinte-like plants in his cross, he argued that as few as five loci might be responsible for much of the phenotypic difference between maize and teosinte. Later work by John Doebley et al. found quantitative trait loci (QTL; see Glossary) consistent with this idea (Doebley and Stec, 1991, 1993), and careful genetic analysis of these regions allowed Doebley's group to dissect some of these QTL down to the responsible genes. The two best-studied cases are the loci *teosinte branched1* (*tb1*) and *teosinte glume architecture* (*tga*)*. tb1* is responsible for the shortening and feminization of the axillary branches in domesticated maize (Doebley et al., 1997; Studer et al., 2011), while *tga* is responsible for the hardened fruitcase of teosinte (Wang et al., 2005). Changes at *tb1* are due to genetic alterations at distant regulatory sequences while critical changes at *tga* are likely to be due to amino-acid changes. Knowledge of these loci provides some of the best examples to date of how human selection for certain physical traits in maize has led to specific changes at the DNA level.

Many other genes, in addition to *tb1* and *tga1*, have played a role in domestication, however. The analysis of genetic diversity from whole-genome sequences of multiple maize and teosinte has identified nearly 500 regions of the genome that show evidence of selection during domestication (Hufford et al., 2012). Intriguingly, some of these regions do not contain genes, and many of the genes identified show differences in expression, pointing to the potentially important role of regulatory variation. Overall, more than 3000 genes show reduced diversity due to the impact of selection. In spite of this strong selection, maize landraces still retain more than 80% of the diversity found in teosinte, and it should be possible to identify potentially useful genetic diversity in landraces or teosinte while maintaining the key genetic contributors to improved maize.

## Genetic diversity and analyzing complex traits

The modern diversity of inbred lines, ranging in their flowering time from 50 to 120 days and in height from 3 to 15 feet tall, derives from maize landraces. Buckler, Holland and McMullen took advantage of this natural diversity to create a widely-used tool, the nested association mapping (NAM) panel, which consists of 5000 recombinant inbred populations (see Glossary) generated by crossing 25 diverse maize lines to the same recurrent parent (McMullen et al., 2009). This population is being used to identify the genes responsible for complex traits, such as flowering time (Buckler et al., 2009), plant architecture (Brown et al., 2011; Tian et al., 2011), and resistance to pests (Kump et al., 2011; Poland et al., 2011). It appears that variation for most traits results from numerous genes that each have a small effect on a trait. Maize differs in this regard from species that are self-pollinated, such as rice, sorghum and *Arabidopsis*, which frequently have fewer loci that produce large effects on traits (Lin et al., 1995; Salome et al., 2011; Huang et al., 2012). Teosinte, as an out-crossing species, is similar to maize in this regard (Weber et al., 2008).

Another powerful approach in maize that takes advantage of its diversity and history of outcrossing is that of genome wide association studies (GWAS), which exploits ancient recombination events that have shuffled and reshuffled genes. GWAS looks for correlations between variation at individual markers and a trait, and has become an important complement to the use of experimental crosses. By using detailed genetic and phenotypic information from the NAM line founders and by comparing this information to genotypic information obtained from their recombinant NAM progeny, we can begin to predict the phenotypic effects produced by the variants of many genes (Wallace et al., 2014). Other collections of maize inbred lines are also available for GWAS studies (Flint-Garcia et al., 2005). Using recently developed, high-throughput sequencing methods, a population of 2815 maize lines was genotyped with more than 600,000 single nucleotide polymorphic markers (Romay et al., 2013). This density of marker information worked well for identifying the genetic basis of simple traits using GWAS, but more markers will be needed for analyzing complex traits.

The eventual goal of these efforts is to link genotypes with phenotypes for use in predictive breeding studies (see Glossary). Predictive breeding will help breeders to create improved crops, by selecting alleles from different inbred maize lines to generate the combination of traits needed in a particular environment.

## Conclusion

Today maize is one of the most important crops worldwide. As we try to increase yields, improve sustainability, and adapt maize to changing environmental and climatic conditions, we can take advantage of our understanding of the natural history of maize and its status as an important model organism. Future efforts at incorporating novel alleles into modern germplasm via selective breeding or transgenics, as well as developing predictive breeding models, will benefit from the history of research in maize, as well as its rich heritage of genetic diversity.
